# Narrow Segment Driven Multistep Magnetization Reversal Process in Sharp Diameter Modulated Fe_67_Co_33_ Nanowires

**DOI:** 10.3390/nano11113077

**Published:** 2021-11-15

**Authors:** Javier García, Jose A. Fernández-Roldán, Roque González, Miguel Méndez, Cristina Bran, Víctor Vega, Silvia González, Manuel Vázquez, Víctor M. Prida

**Affiliations:** 1 Departamento de Física, Facultad de Ciencias, Universidad de Oviedo, C/Federico García Lorca 18, 33007 Oviedo, Spain; fernandezroljose@uniovi.es (J.A.F.-R.); uo237003@uniovi.es (R.G.); uo83049@uniovi.es (M.M.); gonzalezgana@uniovi.es (S.G.); 2 Instituto de Ciencia de Materiales de Madrid, ICMM-CSIC, Sor Juana Inés de la Cruz 3, 28049 Madrid, Spain; cristina.bran@icmm.csic.es (C.B.); mvazquez@icmm.csic.es (M.V.); 3 Laboratorio de Membranas Nanoporosas, Edificio de Servicios Científico Técnicos “Severo Ochoa”, Universidad de Oviedo, C/Fernando Bonguera s/n, 33006 Oviedo, Spain; vegavictor@uniovi.es

**Keywords:** electrodeposition, magnetic nanowires, diameter modulation, FORC, magnetization reversal, MOKE, spintronics

## Abstract

Magnetic nanomaterials are of great interest due to their potential use in data storage, biotechnology, or spintronic based devices, among others. The control of magnetism at such scale entails complexing the nanostructures by tuning their composition, shape, sizes, or even several of these properties at the same time, in order to search for new phenomena or optimize their performance. An interesting pathway to affect the dynamics of the magnetization reversal in ferromagnetic nanostructures is to introduce geometrical modulations to act as nucleation or pinning centers for the magnetic domain walls. Considering the case of 3D magnetic nanowires, the modulation of the diameter across their length can produce such effect as long as the segment diameter transition is sharp enough. In this work, diameter modulated Fe_67_Co_33_ ferromagnetic nanowires have been grown into the prepatterned diameter modulated nanopores of anodized Al_2_O_3_ membranes. Their morphological and compositional characterization was carried out by electron-based microscopy, while their magnetic behavior has been measured on both the nanowire array as well as for individual bisegmented nanowires after being released from the alumina template. The magnetic hysteresis loops, together with the evaluation of First Order Reversal Curve diagrams, point out that the magnetization reversal of the bisegmented FeCo nanowires is carried out in two steps. These two stages are interpreted by micromagnetic modeling, where a shell of the wide segment reverses its magnetization first, followed by the reversal of its core together with the narrow segment of the nanowire at once.

## 1. Introduction

Nowadays, technology is pushing forward, increasing efforts in the scientific community to deeply understand and control nature at lower scales. The design of new concepts based on nanotechnology makes essential the understanding of physical phenomena and the potential effects that spatial confinement may produce on nanostructured materials. The field of magnetism and magnetic materials has been mostly linked to applications in data or energy storage and conversion [1,2]. The pathway that this research field has taken towards nanotechnology is prompted by the need to increase the density of stored information, the increase in the surface to volume ratio in energy exchange systems, or to study and control spin-based phenomena for spintronic applications [3,4,5,6]. Besides the wide range of nanomaterials investigated to date, magnetic nanowires have attracted much attention due to their ability to control their magnetic properties through properly tuning their composition, shape, size, or spatial arrangement [7,8,9]. Within this context, the research focuses on the intrinsic magnetic behavior of single nanowires to explore novel magnetic phenomena arising at nanoscale. The implementation of different characterization techniques, based on magnetotransport, magnetic force microscopy, or magneto-optics, among others, has made this fact possible [10,11,12]. On the other hand, the application concepts often require 3D nano-architectures, which implies a collective magnetic behavior, including magnetostatic or exchange interactions, among nanowires or layers [13,14,15,16]. In such case, techniques such as vibrating sample magnetometry (VSM) or superconducting quantum interference devices (SQUID) help to obtain a wider picture of the collective magnetic behavior of the nanostructured system. However, in both cases, the progress in the field of nanomagnetism has the effect of bringing increasingly complex structures, such as core-shell or compositional and geometrical modulated nanowires, into focus [17,18,19,20,21,22,23,24,25,26]. Such complexity has entailed the employment of different data treatment models such as First Order Reversal Curve (FORC), or the use of micromagnetic simulations, in order to fully understand or decouple the different contributions to the overall magnetic behavior [13,27,28,29,30].

Most of the applications require the control of the reversal magnetization mechanism of the magnetic elements. One of the most researched routes focuses on interfering in the nucleation and propagation of magnetic domain walls through the design of 2D geometrical defects (notches or antinotches) as nucleation or pinning centers [31,32,33]. In 3D cylindrical nanowires, the diameter modulation can induce enough local anisotropy to overcome the exchange interaction at the transition and split the magnetization reversal into two processes [34,35,36]. A key factor needed to achieve such control on the domain wall dynamics is the sharpness of the diameter modulated transition [8]. Moreover, the diameter of the nanowires strongly affects the type of domain wall that the magnetization reversal undergoes through, which may potentiate the retain of the domain wall at the diameter transition [37]. Furthermore, the dependence that FeCo alloys present with respect to the diameter of nanowires, and their tunability on magnetic hardness and saturation magnetization as a function of the alloy composition, makes overall a potential system with which to investigate and control the magnetization reversal at the nanoscale [38]. Moreover, the Fe_2_Co phase stabilizes in a body center cubic crystalline structure with high saturation magnetization and low magnetocrystalline anisotropy, then enforcing the predominant role of shape anisotropy and thus the effect of geometrical modulation.

In this work, the magnetic properties of diameter modulated FeCo nanowires are investigated. Bisegmented nanowires have been electrodeposited in a prepatterned anodic alumina membrane acting as a template. Standard magnetometry supported with FORC measurements were employed to understand the collective magnetic behavior of the nanowire array. In addition, magneto-optical measurements and micromagnetic simulations on single nanowires have been carried out to obtain more detailed information regarding the magnetization reversal of individual diameter modulated FeCo nanowires. 

## 2. Materials and Methods

### 2.1. Bisegmented Nanowires Fabrication

**Figure 1:**
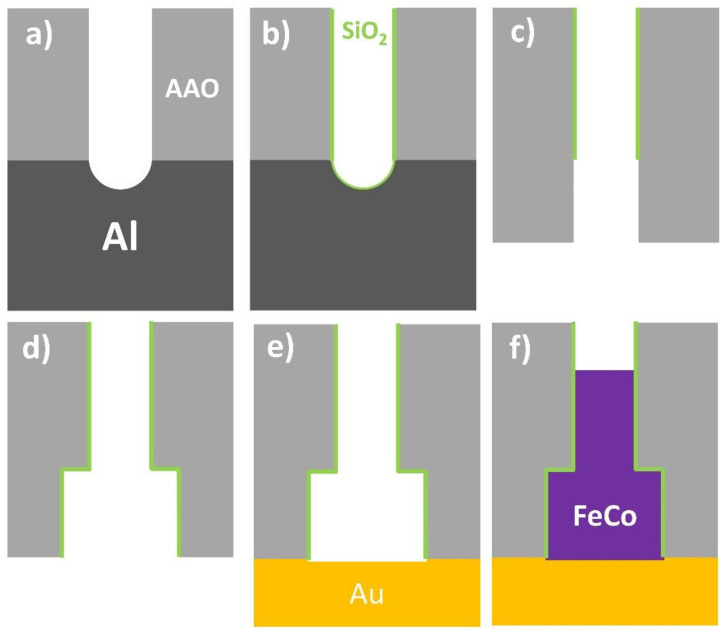
Schematic process flow for the fabrication of bisegmented diameter modulated nanowires embedded into the prepatterned nanopores of anodic aluminum oxide (AAO) template: (**a**) Hard anodization of the Al substrate. (**b**) Coating of AAO surface with a thin SiO_2_ layer by ALD. (**c**) Reanodization of the coated AAO substrate and removal of Al substrate and alumina barrier layer. (**d**) Pore widening and SiO_2_ layer coating. (**e**) Deposition of Au electrode by sputtering and subsequent electrodeposition of Au layer. (**f**) Electrochemical deposition of bisegmented FeCo nanowires inside the patterned pores of the alumina template.

Diameter modulated FeCo nanowires were synthesized by template-assisted pulsed electrodeposition into prepatterned, nanoporous alumina membranes [8,39,40]. High-purity Al foils (99.999%, SMP, Barcelona, Spain), were employed as starting substrates for the synthesis of nanoporous alumina membranes by means of hard anodization (HA) technique [41,42]. The substrates were cleaned in ethanol and isopropanol, and electropolished in a perchloric acid and ethanol (1:3 vol.%) mixture. Afterwards, the hard anodization procedure on starting Al substrates was carried out in 0.3 M oxalic acid electrolyte containing 5 vol.% of ethanol as antifreeze agent, at a temperature of around 0 °C. Prior to HA step, and in order to prevent burning phenomena, the samples were anodized under mild anodization electrochemical conditions at 80 V during 15 min, and the voltage was then swept at 0.04 V/s until reaching the HA regime at 140 V. The HA step lasted 60 min. The nanoporous alumina samples were then submitted to a chemical etching step in phosphoric acid (5 wt.%, 30 °C), which not only increases the pore diameter of the HA nanoporous oxide layer to around 100 nm, but also removes almost completely the protective mild anodization layer at the top of the alumina membranes (Figure 1a) [43].

In order to induce the formation of diameter modulations in the pore channels of HA alumina membranes, a more chemically stable SiO_2_ thin layer was deposited by atomic layer deposition (ALD) technique (Figure 1b) [44]. This layer has been grown by exposing the sample, in a total of 90 cycles, to three different precursors: (3-aminopropyl) triethoxysilane (H_2_N(CH_2_)_3_ Si(OCH_2_CH_3_)_3_) kept at a temperature of 100 °C; water (H_2_O) at 60 °C and ozone (O_3_), while the reaction temperature in the chamber was fixed to 180 °C. According to the estimated deposition rate of 0.06 nm/cycle, the nominal thickness of the SiO_2_ layer corresponds to 4–5 nm. This passivating SiO_2_ thin layer prevents further chemical etching to occur and, therefore, avoids any further enlargement of the pore diameter. The samples were then reanodized under HA conditions (140 V, 0 °C) for 15 min, which causes the growth of a new segment in the alumina nanopores that is not protected by the SiO_2_ layer. Then, the unoxidized Al substrate that remains at the bottom of the alumina membranes was selectively dissolved in CuCl_2_ and HCl solution (Figure 1c). Chemically etching the samples in phosphoric acid (5 wt.%, 30 °C) causes the unprotected alumina pore segment to increase its pore diameter by up to around 250 nm, whereas the one protected by the SiO_2_ ALD layer remains unaltered. After this process, a well-defined and sharp diameter modulation at the interface between the SiO_2_ coated and uncoated pore segments was created (Figure 1d). Finally, the samples were again coated with an SiO_2_ thin layer of around 3.5 nm (70 cycles) in thickness, which avoided corrosion of the metallic nanowires if they were to be released from the HA alumina template [8,43].

In order to perform electrochemical deposition of FeCo alloy, a gold contact was defined in the backside of the samples (Figure 1e) by sputtering and further electrodeposition from a commercial plating bath (Orosene 999, Technic, Lodi, Italy). The electrolyte for FeCo alloy electrodeposition consisted of 0.06 M CoSO_4_, 0.13 M FeSO_4_, and 0.16 M H_3_BO_3_. Continuous N_2_ bubbling was maintained during electrolyte preparation and further electrodeposition processes, with the purpose of avoiding oxidation of Fe^2+^ ions. The pulsed electrodeposition sequence consisted of 3000 pulses of 0.5 s at a constant voltage of −1.8 V measured versus an Ag/AgCl reference electrode, separated by resting pulses of 0.5 s at open circuit potential (Figure 1f).

### 2.2. Characterization Techniques

Morphological and compositional characterization of nanowire samples was carried out in a Scanning Electron Microscope (SEM, JEOL 5600, Akishima, Tokyo, Japan) equipped with an energy dispersive X-ray (EDX) microanalysis system (INCA, Oxford Instruments, Abingdon, UK) while keeping the bisegmented diameter modulated nanowires still embedded into the pores of the alumina matrix. In order to provide more precise measurements of the geometry of the nanowires, free-standing single nanowires were also studied under a Transmission Electron Microscope (TEM, JEOL-2000-EXII, Akishima, Tokyo, Japan). This system has also been employed to obtain Selected Area Electron Diffraction (SAED) patterns of the magnetic FeCo nanowires to evaluate their crystalline structure.

In this work, all magnetic characterizations have been performed at room temperature. In order to have a global picture of the magnetization reversal for the whole system, the alloyed FeCo nanowire arrays measurements were performed in a vibrating sample magnetometer (VSM, Versalab, Quantum Design Inc., San Diego, CA, USA) under applied magnetic fields up to ±3 T. The magnetic study is complemented by characterizing the magneto-optical properties of single bisegmented FeCo nanowires using a NanoMOKE3 (Durham Magneto Optics Ltd., Durham, UK) suited with a quadrupolar magnet that reaches magnetic field values up to ± 1200 Oe. In this case, only the parallel direction with respect to the nanowire long axis has been studied through measuring the longitudinal MOKE signal. To carry out this characterization, the Au back electrode of the sample has been chemically etched with KI+I_2_ solution. Then, the alumina matrix was selectively etched in 0.2 M CrO_3_ and 0.6 M H_3_PO_4_ aqueous solution. After this process, the suspension of the nanowires coated with the SiO_2_ shell protective layer was filtered and filled up with ethanol. Finally, a drop of this suspension is placed and dried on a prepatterned Si wafer, leaving the scattered nanowires parallel to the substrate surface.

### 2.3. First Order Reversal Curve (FORC) Method

The FORC method assumes that the major hysteresis loop presented by a material consists of different hysteretic processes. How these processes magnetically behave depending on the magnetization state of their surroundings is evaluated by sequentially measuring the corresponding minor hysteresis loops. The FORC method theory and measuring protocol can be found elsewhere [27,28,45,46]. To be concise, starting from the saturation state, the applied magnetic field is reduced or reversed down to a certain Hr value, after which the magnetization of the sample is measured on the way back to saturation state. Repeating this protocol for different Hr values in the range between the positive and negative saturation magnetization states, the FORC distribution $\left(\rho \right)$ can be obtained by using the Equation (1),

(1) $$\rho \left(\mathrm{Hr},H\right)=-\frac{\partial^{2}M}{\partial \mathrm{Hr}\partial H}$$

where M is the magnetization and H is the magnetic field applied along the ascendant branch of the minor loop.

This FORC distribution can be represented in a contour plot as a function of the coercivity, Hc, and interaction, Hu, coordinates that gives an idea of the minor loop width (switching field) and its position with respect to the applied magnetic field (interaction). Thus, depending on whether the resulting distribution is elongated, well along the Hc or Hu axes, it may indicate a wide distribution of magnetic elements or a highly interacting system, respectively.

### 2.4. Micromagnetic Simulations of Magnetization Reversal

In order to determine the magnetization reversal mechanism of the bisegmented diameter modulated FeCo nanowires, we have modelled the hysteresis loop and the magnetization reversal process in an individual bisegmented FeCo nanowire made of two cylindrical segments with 2 microns in total length, and with 100 and 200 nm in diameter for the narrow and wide segments, respectively, via micromagnetic modelling with MuMax3 (*mumax^3^,* version 3.10, open source software for micromagnetic simulation; DyNaMat group, Ghent University, Ghent, Belgium) [47]. We have considered typical material parameters of realistic experiments and micromagnetic models of nanowires of FeCo alloys [48,49,50]: a saturation polarization of 2 T, exchange stiffness 25 pJ/m, and a cubic centered magnetocrystalline anisotropy with an anisotropy constant of 10^4^ Jm^−3^. The interpretation of magneto-optical Kerr effect experiments is carried out from the evaluation of the average magnetization in a region of 400 nm × 400 nm size area and 50 nm in depth over the curved surface of the nanowire, in agreement with the typical order of magnitude for the laser penetration depth in the MOKE technique.

## 3. Results

### 3.1. Morphological and Compositional Characterization

**Figure 2:**
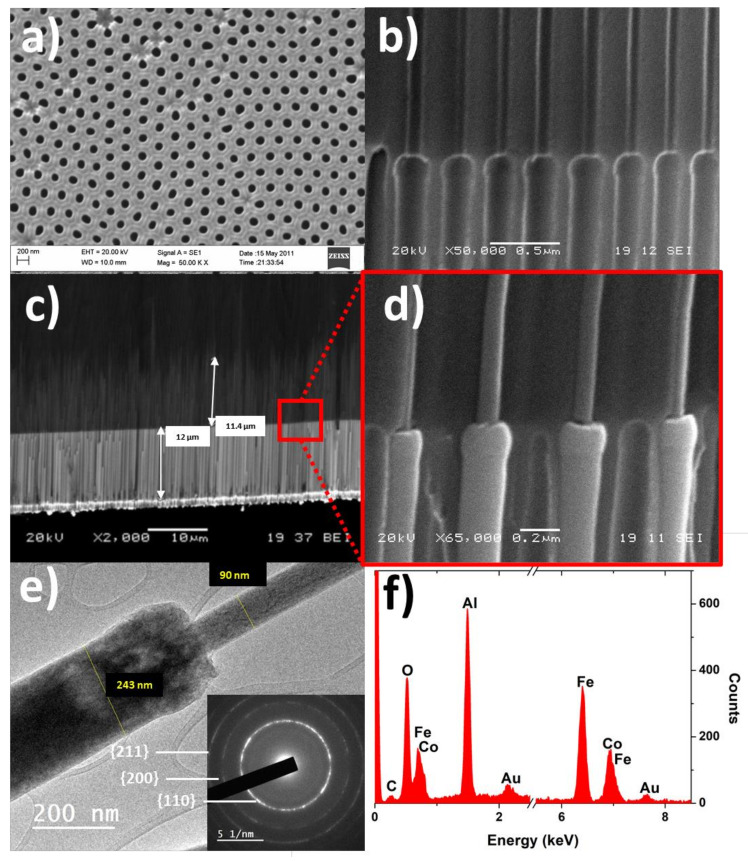
(**a**) SEM micrograph of the patterned nanoporous Al_2_O_3_ membrane top view and (**b**) cross-section. (**c**) SEM cross-section of the electrodeposited nanowires where the lengths of different segments can be estimated. (**d**) Magnified SEM cross-section image of the bisegmented nanowires array at the transition of the diameter modulation. (**e**) TEM image of a diameter modulated Fe_67_Co_33_ nanowire (inset: SAED pattern measured on a single Fe_67_Co_33_ nanowire). (**f**) EDX spectrum performed in the bisegmented FeCo nanowire array embedded into the alumina template.

Assuming a homogeneous composition of the FeCo nanowires along their length, their magnetic response is determined by the geometrical modulation of each nanowire segment, especially the relative difference between the diameters and the sharpness of the transition at the modulation between the segments. In Figure 2, the morphological, microstructural, and compositional characterization of FeCo nanowire samples is summarized.

In Figure 2a,b, the top view and the cross-section of the empty Al_2_O_3_ membrane are shown, respectively. As can be seen in these sub-figures, the nanopores are highly ordered and distributed in a periodic hexagonal arrangement, while geometrically modulated diameter nanopores show a sharp transition between the two pore segments. After the electrodeposition process, the length of the two different nanowire segments can be estimated from Figure 2c, corresponding to 12.0 µm and 11.4 µm for the wide and narrow nanowires segment, respectively. Furthermore, Figure 2d shows a magnified image at the transition between segments of the electrodeposited nanowires. In order to measure more accurately the diameters of each segment, TEM measurements have been carried out (Figure 2e), which results in a wide segment diameter of $\emptyset_{wide}\approx 243\;\mathrm{nm}$ and $\emptyset_{narrow}\approx 90\;\mathrm{nm}$ for the narrower one, respectively. The inset in Figure 2e shows the SAED pattern taken on an isolated nanowire, which is consistent with a cubic *bcc* crystalline structure. Due to the symmetry of a *bcc* lattice, the magnetocrystalline anisotropy becomes negligible, being therefore the magnetic behavior of the nanowires strongly sensitive to their shape and geometrical modulation. Finally, the EDX analysis performed on the nanowire arrays (Figure 2f) sheds an average composition for the electrodeposited nanowires of Fe_67_Co_33_.

### 3.2. Magnetic Properties

**Figure 3:**
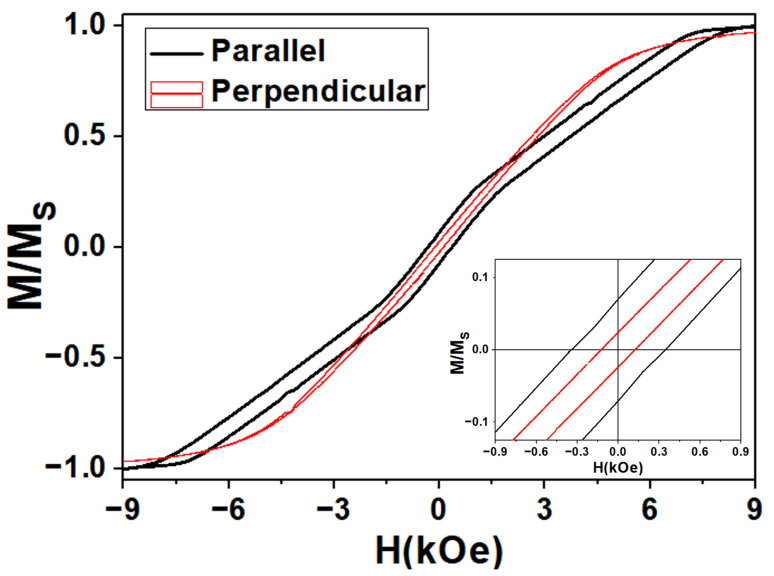
Room temperature hysteresis loops under parallel (black) and perpendicular (red) applied field direction with respect to the nanowires’ long axis. The inset shows a zoom into the low-field region.

Room temperature hysteresis loops (HL) are plotted in Figure 3 under an applied magnetic field that is parallel and perpendicular with respect to the nanowires axis. As observed, the perpendicular HL shows an almost anhysteretic behavior and nearly constant magnetic susceptibility that is typical of a hard magnetization axis. The hysteresis loops along both directions are quite similar in terms of coercivity, remanence, and saturation field. It is clear that the parallel HL is affected by magnetostatic interactions, as is inferred by the tilting of the hysteresis loop along the field axis while keeping its width constant. Such magnetostatic interaction among nanowires typically reduces coercivity as well as remanence while increasing the saturation field, thus reducing the differences between both HL, which are measured along the parallel and in perpendicular directions to the nanowires’ axis [51]. However, the parallel HL shows a more complex magnetic behavior, where the two-step magnetization reversal process is demonstrated through the existence of two different contributions to the magnetic permeability, as can be clearly appreciated. 

**Figure 4:**
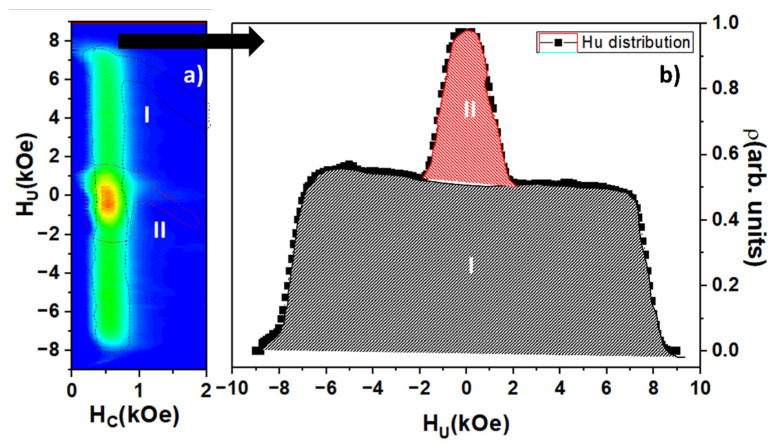
(**a**) FORC diagram of the bisegmented FeCo nanowire array measured along the parallel direction of applied field with respect to the nanowires long axis. Two distributions, I and II, are highlighted as an eye guidance. (**b**) FORC interaction field profile (Hu) where both contributions, I (red) and II (black), are also highlighted.

FORC measurements carried out along the parallel direction of the applied magnetic field with respect to the nanowires’ long axis can provide more information regarding these two different magnetic behaviors. In Figure 4a, the parallel FORC distribution is represented. Looking to the contour plot of FORC distribution, the coexistence of two different distributions appears more clearly, where highly interacting (I) and low interacting (II) distributions can be identified. However, both distributions seem to a present similarly coercive field. Considering that both nanowire segments are made of the same Fe_67_Co_33_ alloy and that the internanowire distance is kept constant, the distance among the edges of the narrow segments is larger than in the case of the wide segments. This fact is in accordance with the settling down of a higher magnetostatic interaction among the wider nanowire segments, which would agree with the FORC distribution I [52]. Furthermore, the existence of these two FORC distributions also points out a two-step magnetization process evidencing that the magnetostatic interactions among both the wider and thinner nanowire segments, respectively, strongly dominate the magnetic behavior of the system instead of the exchange interaction between segments of same nanowire. In Figure 4b, the projection of FORC distribution on the interaction field axis (Hu) is shown, where, once again, the two magnetic behaviors are clearly differentiated. The integration of the whole Hu distribution would be proportional to the net magnetization due to normalization. According to this and integrating separately both FORC distributions, it can be found that the respective contributions to the net magnetization of the distribution I (narrow segment) and distribution II (wide segment) would be of around 14% and 86%, respectively. Comparing with the relative contribution to the total volume of both nanowire segments, that is 12% for the narrow and 88% for the wide segment, the result is in good agreement, considering that this contribution corresponds only with the hysteretic component of the magnetization reversal process and the large dispersion in length of the narrow segment of the nanowires. However, it seems clear that the coercive or switching fields of both FORC distributions are so close that they cannot be distinguished by means of this method. By projecting the FORC distribution onto the Hc axis, the coercive or switching field distribution of the nanowire array with a main coercive field of around 570 Oe and a half width at a half maximum of 240 Oe can be obtained. Although the width of distribution is apparently large, it may contain the switching field distributions of both narrow and wide segments. However, due to the FORC measurement protocol, the potential convolution of the two distributions cannot be easily resolved.

**Figure 5:**
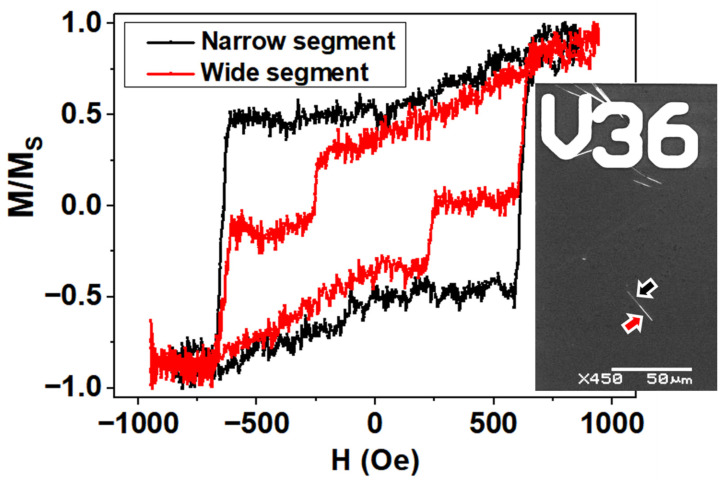
MOKE Hysteresis Loops measured on the narrow (black) and wide (red) segment of single bisegmented FeCo nanowires with the applied magnetic field along the nanowires long axis: The inset shows a SEM image of the measured nanowire highlighting the narrow (black arrow) and wide (red arrow) segments.

These results point out the different magnetic behavior between the wide and narrow nanowire segments and, more importantly, reflect that this peculiar magnetic behavior is followed by most of the nanowires in the sample. This fact ensures that the characterization of the few individual bisegmented nanowires is representative of the sample, which validates the MOKE measurements presented in Figure 5.

To measure the MOKE HL of single isolated bisegmented Fe_67_Co_33_ nanowires, they were dispersed onto the surface of a prepatterned Si substrate in order to identify the measured nanowires. In Figure 5 the MOKE HL corresponding to the narrow and wide segments of a single isolated diameter modulated Fe_67_Co_33_ nanowire are shown. In case of the loop of the narrow segment, the magnetization reversal is carried out homogeneously at a switching field of around 640 Oe. On the other hand, the MOKE HL of the wide nanowire segment always displays a two-step magnetization reversal, being the first one magnetically softer (250 Oe), while the other magnetization jump coincides with the reversal at the same switching field value of the narrow segment of the nanowire.

### 3.3. Micromagnetic Modelling of the Reversal Process and MOKE Model

**Figure 6:**
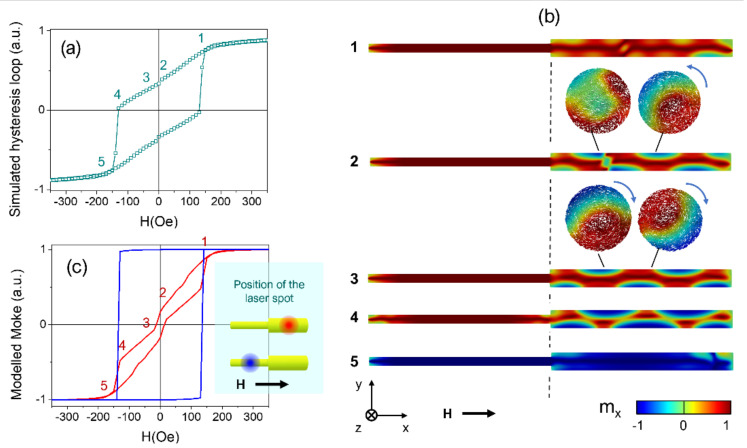
Simulated hysteresis loops of an individual bimodulated Fe_67_Co_33_ nanowire (**a**) and longitudinal cross-sections of the magnetic configurations in the nanowire at the magnetic states labelled from 1 to 5 (**b**). Transverse cross-sections in the nanowire are shown at selected positions. The arrows indicate the circulation (chirality) of the magnetization. Modelled MOKE signal (**c**) from the micromagnetic simulations for the two positions of the laser spot in each respective nanowire segment.

The modelled hysteresis loop of Figure 6a reveals a complex reversal process that begins with gradual changes of magnetization, with the applied field starting from saturation up to remanence (labels from 1 to 2), and an irreversible magnetization jump at small negative applied fields close to remanence (from 2 to 3). A closer inspection of the magnetic configuration in the nanowire displayed in Figure 6b (1) suggests that the magnetization reversal begins with the nucleation of a pair of vortex structures with opposite chirality along the whole length of the wide segment. These vortex structures consist of a core wherein the magnetization is aligned parallel to the nanowire axis (displayed in red color) and a shell wherein the magnetization rotates around the core (also called vortex tubes [23,49,50]). Such vortex structures have been experimentally reported in X-ray magnetic circular dichroism images in wires with notches [35,53] and predicted in wires with periodical modulations in diameter [50]. These vortex tubes are spaced by a topologically non-trivial magnetic domain wall (DW), where the magnetization has a large component perpendicular to the nanowire long axis. As the reverse magnetic field increases in strength, the magnetization in the shell of the wider segment reverses (displayed in blue color) and the tubes gradually transform into a three-dimensional (3D) skyrmion tube state, as depicted in Figure 6b (2). This structure is topologically protected and evidences a corkscrew pinning mechanism that manifests through the helical core of the tube [50]. Importantly, here, the corkscrew pinning is uniquely induced by the single modulation in diameter in contrast to the results in Refs. [49,50]. Secondly, note that the position of the magnetic DW between both skyrmion tubes with opposite chirality is asymmetrically placed inside the wide segment. This asymmetry evidences the preference of such a skyrmion tube, which is on the right part of the nanowire (closer to the wider end), as determined by the redistribution of volume magnetic charges in the wire and surface charges at the single diameter modulation. More broadly, research using controlled models is needed to determine the interplay of the chirality of the skyrmion tubes in these bisegmented nanowires, similar to previous reports [49,50].

At a certain negative magnetic field value, the skyrmion tube on the left side of the wide segment of the FeCo nanowire is annihilated and the skyrmion tube on the right part fills the whole wide segment in Figure 6b (3). A gradual decrease in the magnetic field continues the reversal of the magnetization at the shell of the skyrmion tube in Figure 6b (4) (accompanied by a narrowing of the core displayed in red), manifesting the corkscrew pinning mechanism that dominates the reversal process [7]. During the whole reversal process, the segment with a narrow diameter remains axially magnetized apart from the vortex structure that originated at the left end of the bisegmented diameter-modulated nanowire. The switching process is completed with the simultaneous magnetization switching in both nanowire segments at a certain magnetic field value, for which the core of the skyrmion is sufficiently small to accommodate within the nanowire segment of minor diameter, thus triggering the magnetization reversal of the narrow segment of the nanowire.

### 3.4. Comparison between Experimental and Modelled Magneto-Optical Kerr Effects

The previous discussion on the magnetization reversal process in a single bisegmented diameter modulated FeCo nanowire paves the way for the contrast between the experimental MOKE measurements in Figure 5, with the modelled MOKE loops for a laser spot focused either on the wide or the narrow segment of the nanowire. The squared hysteresis loop displayed in blue in Figure 6c reflects that the narrow segment remains mostly axially magnetized. Therefore, the switching mechanism in the narrow segment is triggered by the magnetization reversal in the wide segment, evinced for a common switching field in both hysteresis loops, in agreement with a corkscrew pinning process.

A closer inspection of the simulated MOKE loop shown in Figure 6c with the laser spot on the wide segment (displayed in red) reveals a gradual change in the magnetization for positive field values that evidences the rotation of the magnetization on the nanowire surface during the formation of the skyrmion tube. Importantly, the magnetization Barkhausen jump close to the remanence in Figure 6c (3) is magnified in the simulated MOKE loop as compared with the micromagnetic model in Figure 6a (3), owing to the surface nature of the technique. As a result, this irreversible magnetization jump in the simulated MOKE loop likely reflects the annihilation of one of the pair of skyrmion tubes.

Considering the signal to noise ratio of the experimental MOKE loops, it is challenging to confirm the particular mechanism by which the wide segment reverses its magnetization through. However, the contrast of the experimental measurements with the simulations indicates that the wide segment magnetization reversal is carried out in two steps, where the magnetically softer one is associated with the shell of the wide segment, and the second jump with its core reversal. Furthermore, the magnetization reversal of the wide segment core triggers the magnetization reversal of the narrow segment, as is confirmed by both MOKE simulations and experiments. It is important to bear in mind that previous reports have concluded that a large saturation magnetization such as the one of Fe_67_Co_33_ and the confinement of the cylindrical geometry are the two main ingredients required to observe curvature-induced states as skyrmion tubes in geometrically modulated nanowires [50,54].

## 4. Conclusions

Controlling the magnetic properties of nanostructures is of great interest for applications based on multistep magnetization reversal and domain wall propagation. Diameter modulations can act as pinning or nucleation centers of magnetic domain walls in 3D nanowires. In this work, sharp diameter modulated Fe_67_Co_33_ nanowires have been electrochemically grown into the pores of prepatterned alumina membranes. Macroscopic magnetometry, as well as FORC analysis, have pointed out the coexistence of two different magnetization reversal contributions which can be associated to the narrow (low magnetostatic interaction) and wide (large magnetostatic interaction) segment. However, when these bisegmented nanowires are isolated, the magnetization reversal mechanism turns out to be more complex, as observed by means of MOKE measurements. While the narrow segment reverses its magnetization at 640 Oe through a single magnetization jump, the wide segment always presents a two-step magnetization reversal process. Complementing the study with micromagnetic simulations, this effect is consistent with the reversal of the magnetization on the wide segment surface at the field value of 250 Oe, while the magnetization of its nucleus is stabilized by the magnetization of the narrow segment, possibly forming skyrmion structures, as inferred by the micromagnetic simulations. When the applied magnetic field is further reversed, the inversion of remaining magnetization of the wide segment core takes place together with the inversion of the narrow segment simultaneously at 640 Oe. This result represents the experimental evidence compatible with the reversal mediated by a corkscrew pinning mechanism in cylindrical nanowires without the need of periodical geometric modulations. In addition, the results shown here suggest that single sharp geometrical modulations are sufficient to induce skyrmion-like structures in cylindrical nanowires with large saturation magnetization. This is of major importance for the development of applications based on 3D nanomagnetism and spintronic applications of topologically non-trivial spin-textures.

## Data Availability

Data presented in this study are available on request from the corresponding author.
